# The Role of the CopA Copper Efflux System in *Acinetobacter baumannii* Virulence

**DOI:** 10.3390/ijms20030575

**Published:** 2019-01-29

**Authors:** Saleh F. Alquethamy, Marjan Khorvash, Victoria G. Pederick, Jonathan J. Whittall, James C. Paton, Ian T. Paulsen, Karl A. Hassan, Christopher A. McDevitt, Bart A. Eijkelkamp

**Affiliations:** 1 Research Centre for Infectious Diseases, School of Biological Sciences, University of Adelaide, Adelaide 5005, South Australia, Australia; salquethamy@student.unimelb.edu.au (S.F.A.); khorvash.m@husky.neu.edu (M.K.); victoria.pederick@gmail.com (V.G.P.); jon.whittall@unisa.edu.au (J.J.W.); james.paton@adelaide.edu.au (J.C.P.); 2 Department of Microbiology and Immunology, The Peter Doherty Institute for Infection and Immunity, University of Melbourne, Melbourne 3000, Victoria, Australia; 3 School of Pharmacy and Medical Sciences, Sansom Institute for Health Research, University of South Australia, Adelaide 5001, South Australia, Australia; 4 Department of Chemistry and Biomolecular Sciences, Macquarie University, Sydney 2109, New South Wales, Australia; ian.paulsen@mq.edu.au; 5 School of Environmental and Life Sciences, University of Newcastle, Callaghan 2308, New South Wales, Australia; karl.hassan@newcastle.edu.au

**Keywords:** bacterial, metal ions, P_1B-1_, P-type ATPases, virulence

## Abstract

*Acinetobacter baumannii* has emerged as one of the leading causative agents of nosocomial infections. Due to its high level of intrinsic and adapted antibiotic resistance, treatment failure rates are high, which allows this opportunistic pathogen to thrive during infection in immune-compromised patients. *A. baumannii* can cause infections within a broad range of host niches, with pneumonia and bacteraemia being associated with the greatest levels of morbidity and mortality. Although its resistance to antibiotics is widely studied, our understanding of the mechanisms required for dealing with environmental stresses related to virulence and hospital persistence, such as copper toxicity, is limited. Here, we performed an in silico analysis of the *A. baumannii* copper resistome, examining its regulation under copper stress. Using comparative analyses of bacterial P-type ATPases, we propose that *A. baumannii* encodes a member of a novel subgroup of P_1B-1_ ATPases. Analyses of three putative inner membrane copper efflux systems identified the P_1B-1_ ATPase CopA as the primary mediator of cytoplasmic copper resistance in *A. baumannii*. Using a murine model of *A. baumannii* pneumonia, we reveal that CopA contributes to the virulence of *A. baumannii*. Collectively, this study advances our understanding of how *A. baumannii* deals with environmental copper toxicity, and it provides novel insights into how *A. baumannii* combats adversities encountered as part of the host immune defence.

## 1. Introduction

*Acinetobacter baumannii* is an opportunistic human pathogen, found predominately in hospitals and aged-care facilities [1]. *A. baumannii* can cause a wide variety of diseases including pneumonia, wound and burn infections, meningitis, and urinary tract infections. While most *A. baumannii* infections are nosocomial, community-acquired *A. baumannii* infections do occur in immune-compromised individuals [2]. *A. baumannii* is attracting significant attention for its increasing antimicrobial resistance, and the global spread of strains resistant to all available antibiotics is imminent. Carbapenem-resistant *A. baumannii* has been placed at the top of the World Health Organization (WHO) critical pathogens list, being in urgent need of new antibiotics [3]. *A. baumannii* is believed to employ a “persist and resist” strategy, wherein the bacterium adapts to unfavourable conditions by processes including biofilm formation, survival from dissociation, and resistance to a range of antimicrobial stresses, including biocides, antibiotics, and metals [4,5,6].

Metals, such as copper, are used as antimicrobials in healthcare settings and agriculture [7,8]. Further, copper has been shown to be recruited by macrophages and neutrophils for its antibacterial activity within the host [9,10,11,12]. Copper toxicity is primarily associated with increased susceptibility to oxidative stress and mismetallation of non-copper metalloproteins [13]. To counteract host-mediated or environmental copper intoxication, bacteria can express a wide range of efflux systems to maintain metal ion homeostasis. These can be classified into three main families, the heavy metal efflux (HME) family (which is a subfamily within the resistance-nodulation-cell division (RND) superfamily), the P-type ATPase family, and the cation diffusion facilitator (CDF) family.

The HME subfamily of transporters facilitates export of metal ions from the cytoplasm and/or periplasm to the extracellular environment, via a proton-antiport driven process [14]. One example of this family is the copper efflux system CusABC, in *Escherichia coli* [15]. The members of the HME family of exporters consist of three proteins, namely, an inner membrane protein (CusA), a periplasmic adaptor protein (CusB), and an outer membrane protein (CusC). Periplasmic copper is delivered to the CusABC system by CusF, a periplasmic copper chaperone [15]. The P-type ATPases are another large family of transport proteins, which are dependent on adenosine triphosphate (ATP) hydrolysis for activity [16]. In Gram-negative bacteria, this family of transporters has been associated with the unidirectional transport of ions, in either an import or efflux capacity, across the cytoplasmic membrane. One of the most well-characterised P-type ATPases is CopA from *Legionella pneumophila*, which effectively exports copper across the cytoplasmic membrane [17]. Members of the CDF family of transport proteins are exclusively involved in metal ion export. Although zinc, cadmium, and cobalt are the primary substrates of CDF proteins [18], studies have shown that some members of this family can provide protection to other metals, including copper [19]. CDF transporters are antiporters and export divalent metal ions in exchange for monovalent cations (H^+^ or K^+^). Bacterial CDFs are ubiquitous, with YiiP from *E. coli* being a well-characterised representative [20]. 

In addition to the three main families of metal ion efflux proteins, there are other copper resistance systems found in Gram-negative bacteria, such as the periplasmic multi-copper oxidase (e.g., CueO from *E. coli*), that oxidizes Cu^1+^ to Cu^2+^ [21]. Further, CopB, an outer membrane protein, is involved in periplasmic copper resistance; however, exactly how this is mediated remains unknown [22]. Also involved in dealing with periplasmic copper stress is the inner membrane protein CopD, which is predicted to import copper into the cytoplasm, a process aided by the periplasmic CopC copper-binding protein [23,24,25]. Cytoplasmic copper stress is sensed by the MerR-family regulator CueR, which has been shown to be responsible for regulation of, for example, *copA* in *E. coli* [26]. In contrast, periplasmic copper stress sensing is believed to be mediated by CopRS, a two-component regulatory system [27].

Recently, candidates for many of the copper resistance mechanisms described here were identified in *A. baumannii* [6,28]. However, their genetic organisation, regulation, contribution to copper tolerance, and in vivo virulence remain to be determined. Here, we provide a comprehensive overview of the *A. baumannii* copper resistome and identify the P-type ATPase CopA as the primary cytoplasmic copper stress resistance determinant. Subsequently, our analyses of a *copA* mutant derivative show that CopA represents a key modulator of oxidative stress resistance and colonisation of the host’s respiratory tract in a murine model of *A. baumannii* infection.

## 2. Results

### 2.1. CopA Provides Copper Resistance in A. baumannii

We first examined possible redundancy of the putative *A. baumannii* copper resistance mechanisms identified on three distinct gene clusters (Figure S1A and Table S1). These bioinformatic analyses revealed that the ABUW_3320–3327 cluster, which is primary associated with the International Clone 1 lineage, harbours multiple genes that share significant similarity to genes positioned on the *A. baumannii* core genome. We found that the putative outer membrane protein CopB (ABUW_3320) and the multi-copper oxidase CueO (ABUW_3321) shared homology with PcoB (72% similarity) and PcoA (75% similarity) from the ABUW_3226–3228 cluster, respectively. Further, CueA (ABUW_3325) shared 67% similarity with CopA (ABUW_2707) (Figure S1B). Subsequent identification of putative regulator binding sites in the *A. baumannii* genome revealed that the putative copper resistance clusters are most likely regulated by distinct metal sensors. We identified a putative CueR binding site in the intergenic region between *copA* and *copZ*, which are part of the ABUW_2706–2708 cluster. Components of the ABUW_3320–3327 cluster were found to harbour putative CopR binding sites, that is, upstream of *copC* and a gene encoding a *copB*-like protein (Figure S1C).

**Figure 1:**
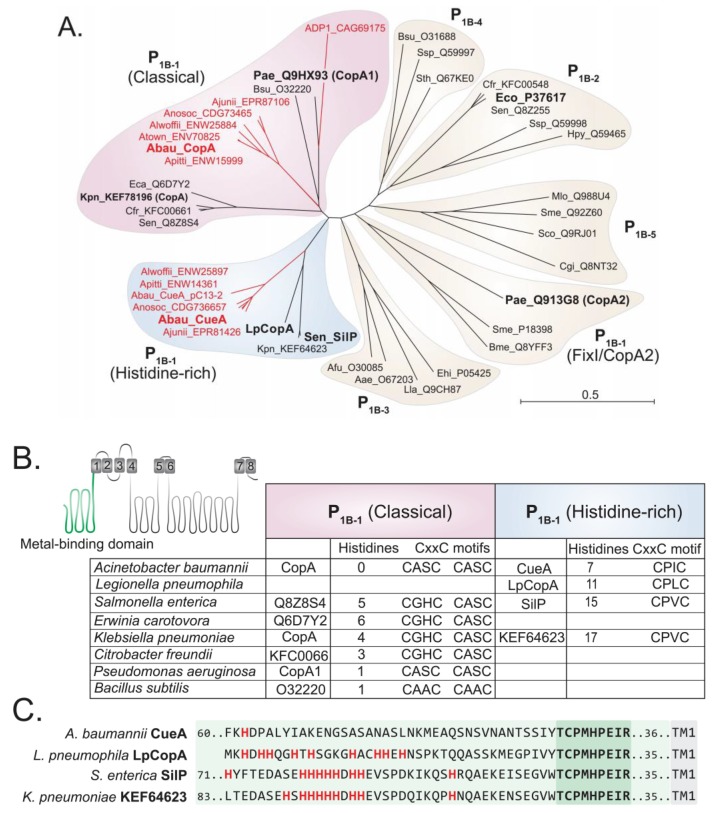
The P_1B-1_ ATPases display significant sequence variation. (**A**) Phylogenetic analysis of the P-type ATPases, including members of the P_1B-1_ (classical, FixI/CopA2, and histidine-rich), P_1B-2_, P_1B-3_, P_1B-4_, and P_1B-5_ subgroups; ADP1, *Acinetobacter baylyi*; Aea, *Aquifex aeolicus*; Afu, *Archaeoglobus fulgidus*; Ajunii, *Acinetobacter junii*; Alwoffii, *Acinetobacter lwoffii*; Anosoc, *Acinetobacter nosocomialis*; Apittii, *Acinetobacter pittii*; Atown, *Acinetobacter towneri*; Bme, *Brucella melitensis*; Bsu, *Bacillus subtilis*; Cgl, *Corynebacterium glutamicum*; Crf, *Citrobacter freundii*; Eco, *Escherichia coli*; Ehi, *Enterococcus hirae*; Hpy, *Helicobacter pylori*; Kpn, *Klebsiella pneumoniae*; Lla, *Lactococcus lactis*; LpCopA, *Legionella pneumophila*; Mlo, *Mesorhizobium loti*; Pae, *Pseudomonas aeruginosa*; Pat, *Pectobacterium atrosepticum*; Sco, *Streptomyces coelicolor*; Sen, *Salmonella enterica*; Sme, *Sinorhizobium meliloti*; Sth, *Symbiobacterium thermophilum*; Ssp, *Synechocystis* sp.; Sty, *Salmonella enterica* Typhy. All P-type ATPases from *Acinetobacter* are depicted in red and those functionally characterised are in a larger font. (**B**) Cartoon model of a P_1B-1_ ATPase is depicted with its eight transmembrane domains. The model serves to highlight the position of the N-terminal metal-binding domain (in green). The table presents data of the N-terminal metal-binding domain of the classical and histidine-rich P_1B-1_ ATPases only, showing the total number of histidine residues and the presence of the CxxC motifs. (**C**) The 40 amino acids at the N-terminal side of the conserved “TCPMHPEIR” domain (dark green shading) of the four histidine-rich P_1B-1_ ATPases are displayed (light green shading). The first transmembrane domain (TM1) starts 35 or 36 amino acids (light green shading) towards the C-terminal end from the “TCPMHPEIR” domain. All histidine residues are displayed in bold and red.

The primary sequences of CopA and CueA indicate that both proteins belong to the P_1B-1_ ATPase family [29]. However, these two sequences, and their orthologs from other *Acinetobacter* strains, clustered into distinct clades of the P_1B-1_ ATPase family (Figure 1A). *A. baumannii* CopA showed the greatest level of similarity to classical P_1B-1_ type ATPases, whereas the *Acinetobacter* CueA proteins formed a distinct and independent clade. In addition to CueA and its orthologs, the *Klebsiella pneumoniae* P_1B-1_ ATPase KEF64623, CopA from *Legionella pneumophila* (LpCopA/Lpg1024) and SilP from *Salmonella typhimurium* also clustered in this clade (Figure 1A).

Further in silico analysis of P_1B-1_ ATPases revealed differences in the amino acid sequences associated with the putative metal-binding domain (MBD) of *A. baumannii* CopA and CueA. The MBD of CopA and the other members of the classical P_1B-1_ ATPases rely primarily upon two CxxC domains (Figure 1B). CueA and the phylogenetically similar proteins (i.e., LpCopA and SilP) were found to only contain a single CxxC domain (Figure 1B). Instead, these phylogenetically distinct proteins have a significantly higher abundance of histidine residues in the MBD, when compared to *A. baumannii* CopA and most of the classical P_1B-1_ ATPases (Figure 1B). Hence, we denoted the CueA clade as the “histidine-rich” P_1B-1_ ATPases. We also found that the MBD of *Acinetobacter* CueA is distinct to LpCopA and SilP, as it lacks a poly-histidine region directly adjacent to a conserved “TCPMHPEIR” sequence (Figure 1C), which accounts for the significantly lower number of histidine residues across the entire MBD (Figure 1B). When examining the phylogenetic clustering of the P_1B-1_ ATPases, excluding the N-terminal MBD, we found that CueA, SilP, and LpCopA still formed their own clade (Figure S2), which illustrates that there are other features within the protein that are distinct between the classical and histidine-rich P_1B-1_ ATPases.

**Figure 2:**
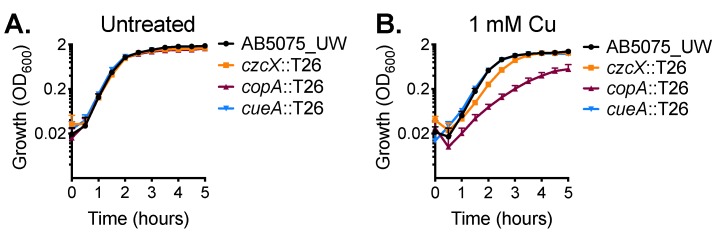
CopA is a contributor to copper resistance in *A. baumannii*. The growth of wild-type (WT) AB5075_UW cells (black) and the *copA*::T26 (burgundy), *cueA*::T26 (blue) and *czcX*::T26 (orange) mutants was determined by measuring the optical density at 600 nm (OD_600_) under (**A**) untreated conditions or (**B**) following supplementation of 1 mM CuSO_4_. The cultures were analysed at 37 °C with shaking (600 rpm), with measurements taken every 30 min. The data represent the mean (± standard error of the mean [SEM]) of at least biological triplicates.

We then sought to experimentally identify the primary contributors for dealing with cytoplasmic copper stress in *A. baumannii* strain AB5075_UW. We analysed the effect of copper stress upon the growth of mutant derivatives of two P-type ATPases, *copA* (ABUW_2707) and *cueA* (ABUW_3325), and a member of the cation diffusion facilitator (CDF) family, *czcX* (ABUW_3226) (Table S2). Mutation of these inner membrane systems alone did not affect growth in standard culturing media (Figure 2A). However, supplementation with 1 mM copper delayed growth of the *czcX*::T26 and *copA*::T26 strains (Figure 2B). Growth of the *czcX*::T26 CDF mutant in the presence of 1 mM copper was perturbed only marginally; however, the *copA*::T26 strain was significantly compromised during logarithmic growth and did not reach the same maximum density as the parental strain. In contrast, the growth profile of the *cueA*::T26 strain closely resembled the parental control.

### 2.2. Functional Characterisation of A. baumannii CopA

**Figure 3:**
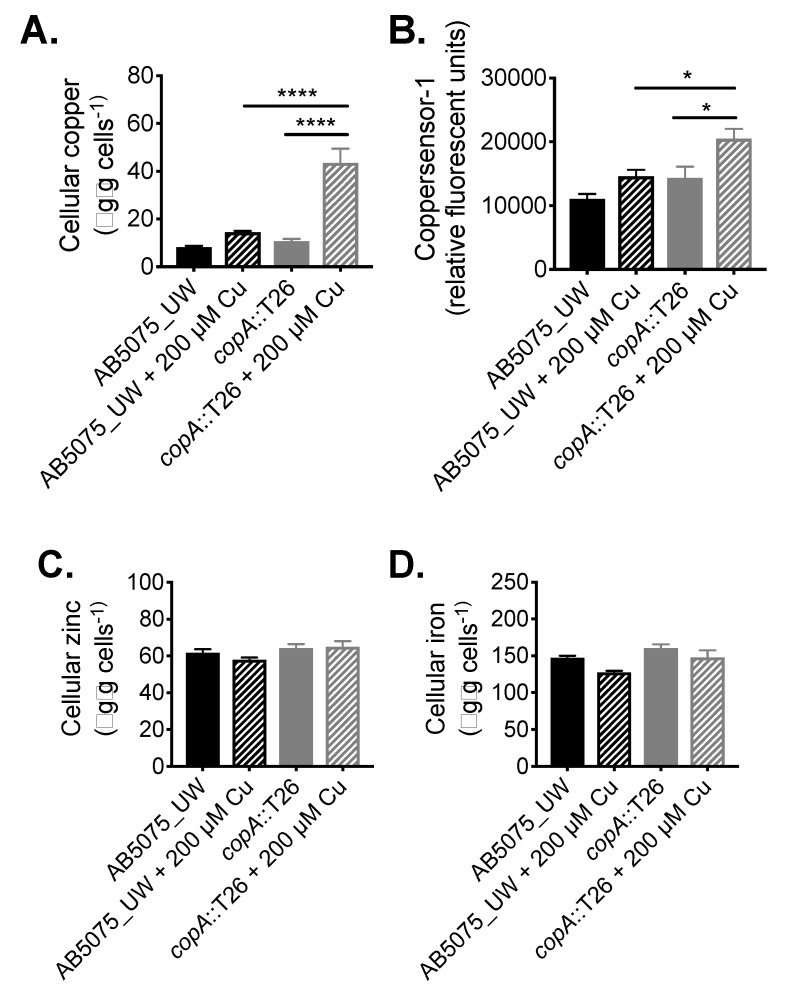
The role of CopA in *A. baumannii* metal ion homeostasis. (**A**) To examine the total cellular copper levels, mid-log phase cells, with or without 200 µM CuSO_4_, were analysed by ICP-MS, with the data represented as the weight of metal (µg) per dry weight of cell material (g). (**B**) Cellular copper was also examined by measuring coppersensor-1 fluorescence by flow cytometry (fluorescent units corrected to unlabelled cells) in cells grown with or without 200 µM CuSO_4_. To examine the total cellular (**C**) zinc and (**D**) iron levels, mid-log phase cells, with or without 200 µM CuSO_4_, were analysed by ICP-MS, with the data represented as the weight of metal (µg) per dry weight of cell material (g). The data in all analyses represent the mean (±SEM) of at least biological triplicates. Statistical analyses were performed with a one-way ANOVA (* *p* < 0.05; **** *p* < 0.0001).

Following its identification as a contributor to copper resistance, we examined the function of CopA in *A. baumannii* in greater detail. The impact of *copA* inactivation upon metal accumulation was analysed by determining the metal content of wild-type (WT) AB5075_UW and *copA*::T26 mutant cells with or without treatment with 200 µM copper, which had no effect on growth of the wild-type cells and induced only minor growth perturbation in the mutant. First, cellular copper levels were determined by inductively coupled plasma mass spectrometry (ICP-MS), which revealed that the *copA*::T26 mutant accumulated 3-fold (*p* < 0.0001; one-way analysis of variance [ANOVA]) more copper compared to the parental strain under copper stress (Figure 3A). Complementing the ICP-MS, we examined intracellular copper levels using coppersensor-1 labelling and flow cytometry. Coppersensor-1 fluorescence was significantly higher (1.4-fold; *p* < 0.05; one-way ANOVA) in the *copA*::T26 mutant compared to the parental strain following treatment with 200 µM copper (Figure 3B). Metal ion intoxication can perturb the homeostasis of other transition metals [28]. Therefore, we analysed the cellular iron and zinc levels by ICP-MS, but no significant differences were observed upon copper treatment (Figure 3C,D).

**Figure 4:**
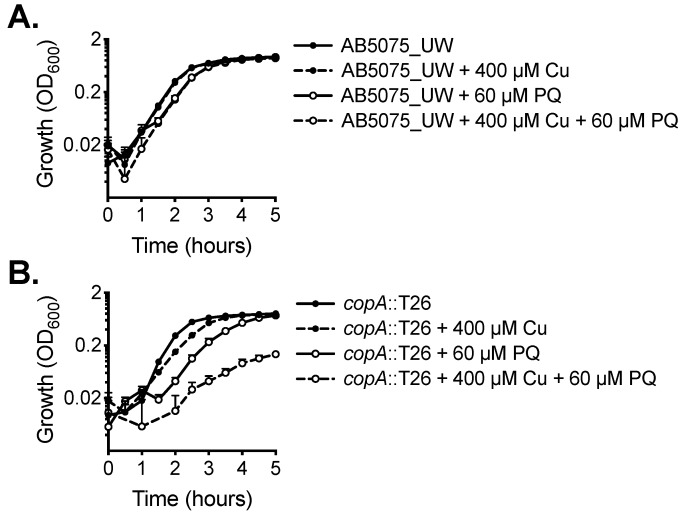
The effect of copper on oxidative stress tolerance. The growth of (**A**) wild-type AB5075_UW cells and (**B**) the *copA*::T26 mutant was determined by measuring the optical density at 600 nm (OD_600_) under untreated conditions or following supplementation of 400 µM CuSO_4_ and/or 60 µM paraquat (PQ). The cultures were analysed at 37 °C with shaking (600 rpm), with measurements taken every 30 min. The data represent the mean (± SEM) of at least biological triplicates.

Copper can exert increased toxicity under conditions of oxidative stress. Accordingly, we examined the impact of copper treatment combined with paraquat, which induces the production of intracellular superoxide stress. The wild-type did not show increased susceptibility to paraquat upon copper stress (Figure 4A). However, growth of the *copA*::T26 mutant was perturbed significantly by the supplementation of paraquat alone, a phenotype even more dramatic upon the addition of copper (Figure 4B).

Overall, our analyses here have identified the P-type ATPase CopA (ABUW_2707) as a key contributor to copper export from the cytoplasm, thereby aiding copper resistance and subsequently protection against oxidative stress.

### 2.3. CopA Plays A Role in A. baumannii Host Colonisation

**Figure 5:**
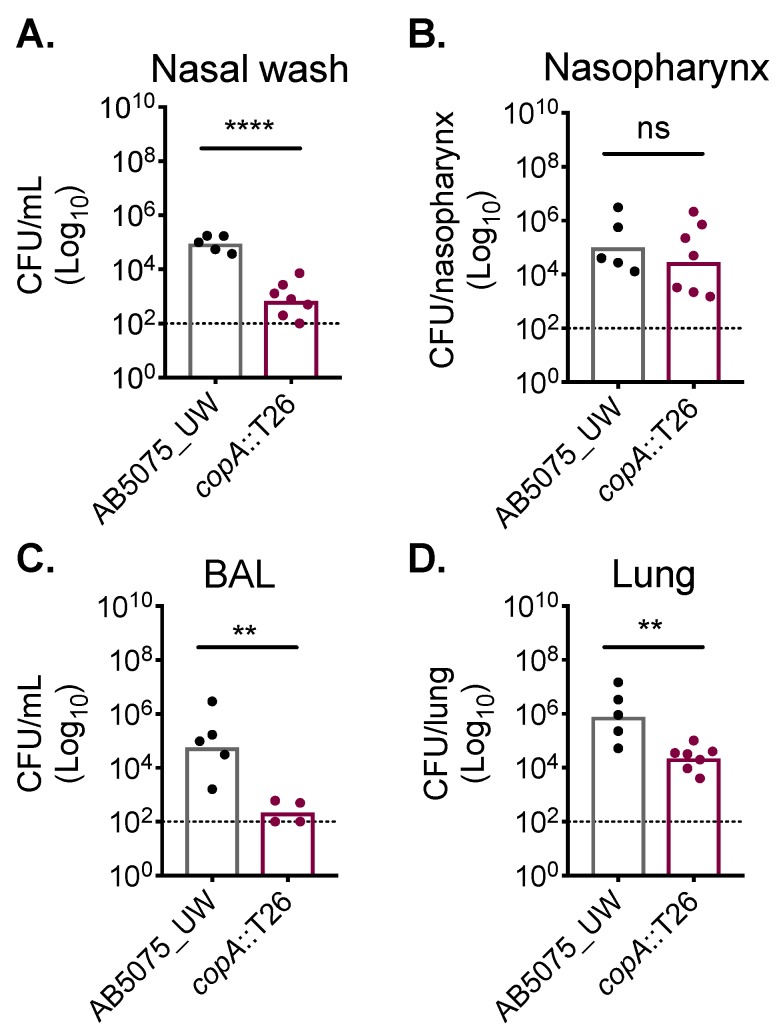
CopA aids in *A. baumannii* murine colonisation. Outbred Swiss mice (5-week-old females), were intranasally challenged with 2 × 10^8^ colony-forming units (CFUs) of strains AB5075_UW (black dots) or its *copA*::T26 derivative (burgundy dots). After 24 h, mice were euthanised and the *A. baumannii* cells were enumerated in (**A**) the nasal wash, (**B**) the nasopharynx, (**C**) the bronchoalveolar lavage (BAL), and (**D**) the lungs. The data represent the geometric mean with the dotted line indicating the limit of detection. Statistical analyses were performed with a Student’s *t*-test (ns = not significant, ** *p* < 0.01; **** *p* < 0.0001).

Copper has been shown to be used as an antimicrobial by the innate immune system to combat bacterial pathogens, such as *Pseudomonas aeruginosa* [30]. However, the role of copper intoxication during *A. baumannii* infection has not been previously examined. Here, we utilised the *copA*::T26 mutant to ascertain whether cytoplasmic copper stress occurs during infection. To mimic a common route of *A. baumannii* infection that leads to significant morbidity and mortality, outbred Swiss mice (female) were challenged intranasally, resulting in the development of pneumonia. At 24 h post-challenge, mice challenged with the AB5075_UW wild-type strain or its *copA*::T26 mutant derivative were euthanised and the bacterial burden in a diverse range of niches was examined (Figure 5). In the upper respiratory tract, we found that the *copA*::T26 mutant was compromised in its ability to colonise the nasopharyngeal lining, as levels of the *copA*::T26 mutant were nearly 60-fold lower than those for the wild-type in the nasal wash (Student’s *t*-test; *p* < 0.0001) (Figure 5A). Interestingly, the colonisation of the nasopharyngeal tissue was not significantly different between the two strains (Figure 5B). CopA was found to play a significant role in *A. baumannii* colonisation of the lower respiratory tract, as mutant colonisation was reduced by more than a 3 log_10_-fold change (Student’s *t*-test; *p* < 0.01) in the bronchioalveolar lavage and in the lung tissue by more than a 2 log_10_-fold change (Student’s *t*-test; *p* < 0.01) (Figure 5C,D). Thus, these findings highlight the major contribution of CopA in *A. baumannii* copper resistance and virulence.

## 3. Discussion

In this study, we examined *A. baumannii* copper resistance by bioinformatically analysing the putative cytoplasmic and periplasmic copper homeostasis mechanisms. This study has provided new insight into the relative roles of the CueR and CopRS regulators in *A. baumannii* copper resistance. We also found that various *A. baumannii* copper resistance mechanisms were positioned on mobile genetic elements, including the ABUW_3320−3327 cluster, and at least one additional cluster encoded on plasmid pC13-2, which shares a high level of homology to ABUW_3320–3327 [31]. Our work has emphasised that *A. baumannii* uses a combination of copper resistance systems that are, at least in part, distinct to that observed in other previously studied Gram-negative bacteria, such as *E. coli* and *P. aeruginosa*. The Cus HME transport system provides *E. coli* with copper resistance during anaerobic conditions [32]; therefore, the lack of a commonly expressed copper HME efflux system in *A. baumannii* may be due to it being a strictly aerobic bacterium. Further, the absence of CopR binding sites in the *pcoAB* cluster of *A. baumannii*, and positioning of *copRS* on the accessory genome, has highlighted that regulation of PcoAB-mediated periplasmic copper resistance in *A. baumannii* requires further examination.

We also showed, for the first time, that the chromosomally-encoded, and highly conserved, P_1B-1_ ATPase CopA provides a high level of copper resistance and protects the cell against oxidative stress. The in vivo significance of our findings was shown by impairment of the *copA*::T26 mutant in the bronchoalveolar lavage (BAL), nasal wash and, to a lesser extent, the lungs. This may be due to copper being an important antimicrobial at mucosal surfaces. An alternative explanation for the difference between the wild-type and *copA*::T26 mutant in colonisation may centre around differential clearance by phagocytic cells. Phagocytic cells have been previously demonstrated to harness the antimicrobial activity of copper to kill bacterial pathogens [28]. Accordingly, the differences seen between the wild-type and *copA*::T26 mutant strains may be a result of their resistance to copper-prosecuted phagocytic killing. Interestingly, the analysis of a putative copper acquisition system, OprC, has previously revealed the significance of copper acquisition in *A. baumannii* virulence, including colonisation of lung tissue [33]. Combined, this highlights the importance of both import and efflux to provide *A. baumannii* with a functional copper homeostasis system during infection.

Through comparative genomics and subsequent copper susceptibility analyses, we have previously speculated that the ABUW_3320–3327 cluster does not play a critical role in copper resistance, as most strains of *A. baumannii* were affected by copper to a similar extent, regardless of the presence of this cluster [28]. In line with these findings, our analyses of a *cueA*::T26 P_1B-1_ ATPase mutant (ABUW_3325) did not reveal a role in copper tolerance. Considering the role of all other members of the ABUW_3320–3327 cluster in dealing with periplasmic copper stress, this observation is not surprising, as CueA-mediated transport of copper back into the periplasm following its removal by CopD would be unanticipated. A factor that should be considered when comparing the functional relevance of *A. baumannii* CopA and CueA P_1B-1_ ATPases is the presence of the copper chaperone *copZ* in the same genetic cluster as *copA*, but not *cueA*, which may contribute to their relative copper efflux efficiencies.

Further bioinformatic analyses revealed that CueA of *A. baumannii* belongs to a distinct group of P_1B-1_ ATPases, which we denoted the “histidine-rich” subgroup. P_1B-1_ ATPases with an elevated number of histidine residues have been previously described in the analysis of P-type ATPases from the Rhizobiales order, which includes nitrogen-fixing plant symbionts, such as the *Sinorhizobium* species, and the human pathogens of the *Bartonella* species [34]. Although none of the histidine-rich P_1B-1_ ATPases from *Sinorhizobium* species have been functionally characterised, the roles of LpCopA and SilP in metal resistance illustrate that the histidine-rich P_1B-1_ ATPases can fulfil important roles. Examination of the key metal-binding residues, as identified in the LpCopA structure, did not reveal any differences between members of the classical and histidine-rich P_1B-1_ ATPases, with the sole exception of LpCopA M711 [17]. We found M711 to be distinct between various species, even within members of the two P_1B-1_ subgroups, which indicates M711 may not play a critical role in the final copper extrusion step by LpCopA.

Overall, this study highlights the significance of cytoplasmic copper toxicity in *A. baumannii* and how it overcomes this to allow expression of its full host-colonisation potential. Further, we have provided novel insights into the variation seen within bacterial P_1B-1_ ATPases. Our analyses also show that CopA and CueA are not functionally redundant, as predicted by bioinformatic analyses.

## 4. Materials and Methods

### 4.1. Bacterial Strains, Chemicals, Media, and Growth

The strains used in this study were purchased from the Manoil Lab (Seattle, WA, USA) *Acinetobacter baumannii* AB5075_UW mutant collection (Table S2). All chemicals were purchased from Sigma-Aldrich (St. Louis, MO, USA), unless otherwise indicated. *A. baumannii* strains were routinely grown in Luria Bertani broth (LB), containing 1% tryptone (BD Bacto), 0.5% yeast extract (BD Bacto), and 1% sodium chloride. For routine overnight culturing of *A. baumannii* strains, a single colony from LB agar was used to inoculate 4 mL of LB medium. Overnight cultures were diluted to an optical density at 600 nm (OD_600_) of 0.01 in either 200 μL for growth assays or 20 mL for all other analyses. For growth assays, cultures in LB media were incubated at 37 °C with shaking in a FLUOStar Omega Spectrophotometer (BMG Labtech, Ortenberg, Germany), with the OD_600_ values presented. The 20 mL cultures used for all other analyses were incubated at 37 °C in an Innova 40R shaking incubator (Eppendorf, Hamburg, Germany) at 230 rpm until they reached mid log-phase (OD_600_ = 0.7). Results are the mean (± SEM) of at least three independent experiments.

### 4.2. Cellular Metal Ion Content Analysis

Total cellular metal analyses were performed as described previously [28]. Untreated and metal-stressed bacteria (LB supplemented with 200 μM CuSO_4_) were harvested at mid log-phase and washed, by resuspension and centrifugation at 7000× *g* for 8 min, three times with phosphate-buffered saline (PBS) containing 5 mM ethylenediaminetetraacetic acid (EDTA), and then three times with PBS. Bacterial pellets were desiccated at 95 °C overnight. The dry cell weight was measured, and the pellets resuspended in 35% HNO_3_ and boiled at 95 °C for 1 h prior to removal of debris by centrifugation. Samples were diluted to a final concentration of 3.5% HNO_3_ and analysed by inductively coupled plasma mass spectrometry (ICP-MS) on an Agilent 7500cx ICP-MS (Adelaide Microscopy, University of Adelaide, Adelaide, SA, Australia). To examine cellular copper levels using coppersensor-1 [35], untreated and metal-stressed bacteria (LB supplemented with 200 μM CuSO_4_) were harvested at mid log-phase and washed, by resuspension and centrifugation at 7000× *g* for 8 min, three times with PBS. Cells were incubated with 5 μM coppersensor-1 for 30 min and washed three times with PBS. The fluorescence of at least 10,000 events was examined on an LSR II flow cytometer (Becton Dickinson, Franklin Lakes, NJ, USA), with the average fluorescence of the positive population calculated. Results for both experiments are the mean (±SEM) of at least three independent experiments, with the statistical significance determined using a one-way ANOVA.

### 4.3. Animal Experiments

Outbred female Swiss mice were anaesthetized by intraperitoneal injection of pentobarbital sodium (Ilium, Laboratory Animal Services, University of Adelaide, Adelaide, SA, Australia) at a dose of 66 μg.g body weight^-1^ [36,37], followed by intranasal administration of 40 μL bacterial suspension containing approximately 2 × 10^8^ colony forming units (CFUs). The challenge dose was confirmed retrospectively by serial dilution and plating. For determination of bacterial loads, mice were euthanized by CO_2_ asphyxiation at 24 h post-challenge. The lungs and nasopharynx were lavaged through the trachea, both with 1 mL sterile PBS. Pulmonary vasculature was perfused by infusion of sterile PBS through the heart, and lungs were subsequently excised. Lastly, the nasopharynx/upper palate was excised (nasopharyngeal tissue). Tissues were homogenized using a Precellys homogeniser (Bertin Instruments, Rockville, MD, USA) and all samples were serially diluted and plated for bacterial counts.

All procedures performed in this study were conducted with a view to minimizing the discomfort of the animals, and used the minimum numbers to generate reproducible and statistically significant data. All experiments were approved (13 September 2016) by the University of Adelaide Animal Ethics Committee (Animal Welfare Assurance number A5491-01; project approval number S-2016-108) and were performed in strict adherence to guidelines dictated by the Australian Code of Practice for the Care and Use of Animals for Scientific Purposes.

### 4.4. Bioinformatics

The *A. baumannii* copper resistance mechanisms were identified using homologous proteins from *P. aeruginosa* and *E. coli* using blastp searches (E-value < 1 × 10^−30^) in NCBI (Table S1). Orthologous genes/proteins across the *Acinetobacter* genomes and between distinct genera were aligned using Clustal Omega. Protein phylogeny was examined using the Neighbor Joining method with Jukes–Cantor distance measurements (1000 bootstrap replicates), all as integrated components of CLC Sequence Viewer 8.0 (Qiagen, Hilden, Germany).

Protein localisation was predicted by SignalP 4.1 [38], and the presence of alpha-helical and beta-sheet transmembrane domains using Minnou [39] and the Prediction of TransMembrane Beta-Barrel proteins tool (PRED-TMMB) [40]. The putative CopR and CueR binding sites were examined as described previously [41,42,43,44]. CopR and CueR binding sequences from *P. aeruginosa* [27,45] were aligned using Clustal Omega, and a scoring matrix was generated using HMMER2 [46]. Following calibration, binding sites were identified in the AB5075_UW genome, using HMMER2 as an integrative part of UGENE v1.18.0 (Unipro, Novosibirsk, Russia).

## Supplementary Materials

Supplementary materials can be found at http://www.mdpi.com/1422-0067/20/3/575/s1.

## Abbreviations

BAL	Bronchioalveolar lavage
CDF	Cation diffusion facilitator
CFU	Colony-forming unit
ICP-MS	Inductively coupled plasma mass spectrometry
HME	Heavy metal efflux
RND	Resistance-nodulation-cell division
WT	Wild-type
