# Supplementary material for: The Role of the CopA Copper Efflux System in Acinetobacter baumannii Virulence
Source: Int J Mol Sci. 2019 Jan 29;20(3):575. doi: 10.3390/ijms20030575 (PMC6387184; doi:10.3390/ijms20030575)
Supplement: Supplementary file 1 [file ijms-20-00575-s001.pdf]

Supplementary Materials

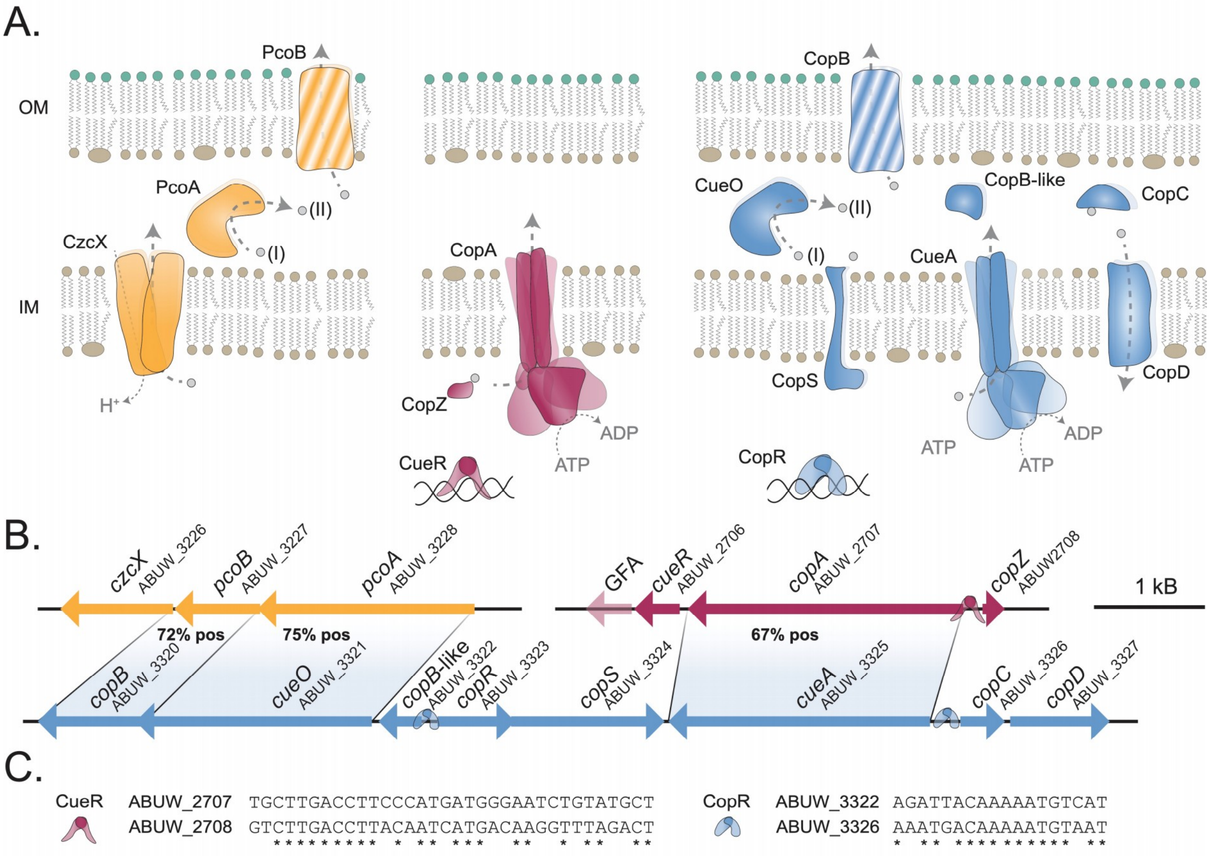

Figure S1. The copper homeostasis mechanisms of *A. baumannii*. (A) Cartoon depiction describing the results of the bioinformatic analyses of the three distinct clusters harbouring putative copper resistance mechanisms in *A. baumannii*; ABUW_3226-28 (yellow), ABUW_2705-08 (burgundy) and ABUW_3320-27 (blue). The inner membrane (IM) and outer membrane (OM) are depicted as phospholipid bilayers, copper is indicated in grey spheres and the direction of transport by dashed arrows. The periplasmic localisation of proteins was determined by SignalP 4.0 [1] and the presence of transmembrane domains by Minnou (http://minnou.cchmc.org) and PRED-TMMB [2]. **(B)** Protein homology presented as the percentage of similar amino acids (% pos) was determined by alignment in blastp. **(C)** The two binding sites for CopR and CueR were identified following the generation of a scoring matrix of known sequences from *E. coli* or *P. aeruginosa*, using HMMER2 [3]. The asterisks indicate conserved base-pairs. The genetic clusters were drawn to scale, using GenBank file CP008706 as a template in UGENE v1.18.0 (Unipro).

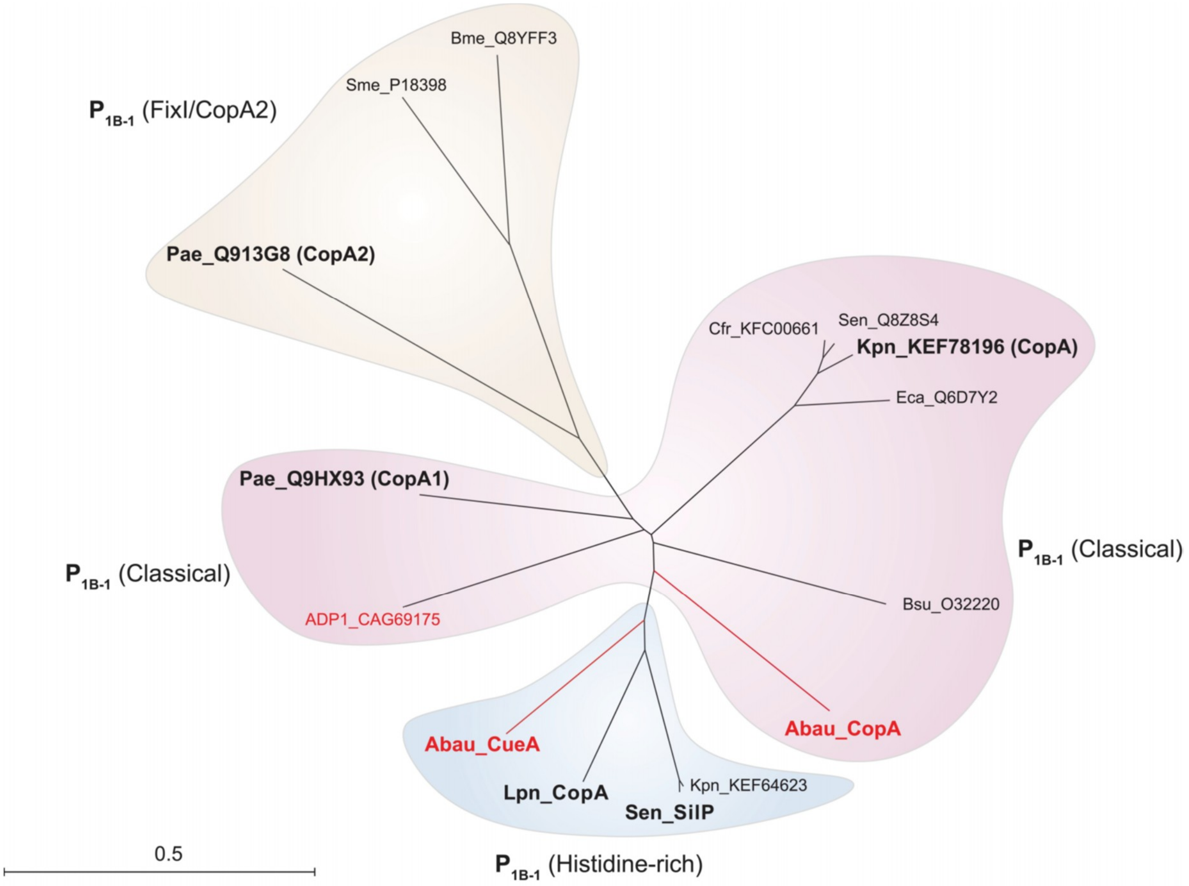

Figure S2. Phylogenetic analysis of the P_{1B-1} ATPases without the amino-terminal metal binding domain. Phylogenetic analysis of the P-type ATPases, including members of the Classical, FixI/CopA2 and histidine-rich subgroups of P_{1B-1} ATPases. ADP1, *Acinetobacter baylyi*; Bme, *Brucella melitensis*; Bsu, *Bacillus subtilis*; LpCopA, *Legionella pneumophila*; Pae, *Pseudomonas aeruginosa*; Pat, *Pectobacterium atrosepticum*; Sen, *Salmonella enterica*; Sme, *Sinorhizobium meliloti*. All P-type ATPases from *Acinetobacter* are depicted in red and those functionally characterised are in a larger font.

1. Petersen, T. N.; Brunak, S.; von Heijne, G.; Nielsen, H., SignalP 4.0: discriminating signal peptides from transmembrane regions. *Nat Methods* **2011,** 8, (10), 785-6.
2. Cao, B.; Porollo, A.; Adamczak, R.; Jarrell, M.; Meller, J., Enhanced recognition of protein transmembrane domains with prediction-based structural profiles. *Bioinformatics* **2006,** 22, (3), 303-9.
3. Finn, R. D.; Clements, J.; Eddy, S. R., HMMER web server: interactive sequence similarity searching. *Nucleic Acids Res* **2011,** 39, (Web Server issue), W29-37.

Table S1. Homologous proteins in *E. coli* and *P. aeruginosa*.

Protein	*A. baumannii*		*E. coli*		*P. aeruginosa*	
Inner membrane P1b-1-type ATPase copper efflux system	ABUW_3325 (CueA)	ABUW_2707 (CopA)	CopA		CopA1 (CueA)	CopA2
Outer membrane beta barrel copper (efflux)	ABUW_3320 (CopB)	ABUW_3227 (PcoB)	PcoB		PcoB	
Copper oxidase	ABUW_3321 (CueO)	ABUW_3228 (PcoA)	CueO	PcoA	PcoA	PA2807
Probable copper-binding protein	n.i.		PcoE		n.i.	
TCRS (Response Regulator)	ABUW_3323 (CopR)		CusR		CopR	
TCRS (Sensor Kinase)	ABUW_3324 (CopS)		CusS		CopS	
Periplasmic sequestering protein	ABUW_3326 (CopC)		CopC		n.i.	
Inner membrane copper (uptake)	ABUW_3327 (CopD)		CopD		n.i.	
MerR	ABUW_2706 (CueR)		CueR		CueR	
Hypothetical periplasmic copper binding protein	ABUW_3322 (CopB-like)		n.i.		n.i.	
Cytoplasmic copper chaperone	ABUW_2708 (CopZ)		n.i.		CopZ1	CopZ2
CDF	ABUW_3226 (CzcX)		n.i.		n.i.	
HME - IMP	n.i.		CusA		CusA	
HME - MFP	n.i.		CusB		CusB	
HME - OMP	n.i.		CusC		CusC	
HME - Chaperone	n.i.		CusF		n.i.	

n.i. = not identified.

Table S2. Strains included in the study.

Name	Locus-tag	Details	Origin
AB5075_UW	Not applicable	Wild-type	
copA::T26	ABUW_2707	tnab1_kr130904p03q144	[1]
czcX::T26	ABUW_3226	tnab1_kr121203p02q112	[1]
cueA::T26	ABUW_3325	tnab1_kr130904p03q191	[1]

1. Gallagher, L. A.; Ramage, E.; Weiss, E. J.; Radey, M.; Hayden, H. S.; Held, K. G.; Huse, H. K.; Zurawski, D. V.; Brittnacher, M. J.; Manoil, C., Resources for genetic and genomic analysis of emerging pathogen *Acinetobacter baumannii*. *J Bacteriol* **2015,** 197, (12), 2027-35.
